# Progress of the Application of Mesoporous Silica-Supported Heteropolyacids in Heterogeneous Catalysis and Preparation of Nanostructured Metal Oxides

**DOI:** 10.3390/ma3020764

**Published:** 2010-01-27

**Authors:** Yuanhang Ren, Bin Yue, Min Gu, Heyong He

**Affiliations:** Department of Chemistry and Shanghai Key Laboratory of Molecular Catalysis and Innovative Materials, Laboratory of Advanced Materials, Fudan University, Shanghai 200433, China

**Keywords:** mesoporous silica, heteropoly acid, polyoxometalates, heterogeneous catalysis, nanostructured metal oxide

## Abstract

Mesoporous silica molecular sieves are a kind of unique catalyst support due to their large pore size and high surface area. Several methods have been developed to immobilize heteropolyacids (HPAs) inside the channels of these mesoporous silicas. The mesoporous silica-supported HPA materials have been widely used as recyclable catalysts in heterogeneous systems. They have shown high catalytic activities and shape selectivities in some reactions, compared to the parent HPAs in homogeneous systems. This review summarizes recent progress in the field of mesoporous silica-supported HPAs applied in the heterogeneous catalysis area and preparation of nanostructured metal oxides using HPAs as precursors and mesoporous silicas as hard templates.

## 1. Introduction

In the last decades, heteropolyacids (HPAs) and related polyoxometalate compounds have attracted much attention as economically and environmentally friendly catalysts [1,2,3,4,5,6,7,8]. HPAs have very strong Brönsted acidity, approaching the superacid range and they are also efficient oxidants. HPAs are very soluble in polar solvents such as water, alcohols, ketones, *etc.* Therefore, HPAs are employed in homogeneous systems as acid and oxidation catalysts and, particularly, they show higher catalytic activity than mineral acids [2,6,9]. On the other hand, HPAs are nontoxic and mildly to non--corrosive, so they are generally recognized as clean and safe catalysts.

Although homogeneous catalytic processes are efficient for a wide variety of reactions, they have some disadvantages. The difficulty in separation of catalyst from the product has led to economical and environmental problems, which is also inconvenient in continuous production. To solve these problems, scientists have mainly explored three different ways to prepare heterogeneous HPA catalysts. The first preparation method is to prepare water-insoluble HPA salts by partial substitution of acidic protons with Cs^+^, K^+^, NH_4_^+^, *etc.* [10,11]. These HPA salts exhibit microporous/mesoporous structures and larger surface area than the pure acids [12,13]. The acidic Cs^+^ salt, Cs_2.5_H_0.5_PW_12_O_40_, was demonstrated as an excellent solid catalyst for various reactions [12,14]. The second method is to support HPAs on acidic or neutral supports such as SiO_2_ [15,16], TiO_2_ [17], ZrO_2_ [18,19], acidic ion-exchange resin [20], or active carbon [21]. Impregnating HPAs on these supports significantly increases the specific surface area, which is very important for heterogeneous catalysis processes. The supports also influence the acidity and catalytic activity of HPAs. It has been found that the basic solids like MgO [3,15] and Al_2_O_3_ [22] tend to decompose the HPAs, causing a significant decrease in their catalytic activities. Silica is the most often used support since it is relatively inert towards HPAs. The third method is to introduce HPAs on mesoporous silica molecular sieves. It has long been a challenge to incorporate HPAs into zeolites to carry out shape-selective catalysis as the pore size of zeolites is normally too small to accommodate large HPA molecules (>1.2 nm). Since the report of the mesoporous silica molecular sieves, such as MCM-41 [23,24], SBA-15 [25,26], and HMS [27,28], mesoporous materials have attracted much attention for their potential applications in the fields of catalysis, functional materials, and nanodevices [29,30,31]. They have uniform channels, large pore size, high specific surface area and high thermal stability. Mesoporous silica molecular sieves are considered to be ideal supports for HPAs compared with zeolites. The pores are large enough for HPA molecules to enter their mesoporous channels. Loading of HPAs on mesoporous silica not only allows transfer of HPA-catalysed reactions from homogeneous to heterogeneous systems to avoid the difficulty in catalyst separation, but also effectively increases the surface area of HPAs [32]. The large pore size of mesoporous silica has a high mass transfer efficiency, which benefits the reactions involving large organic molecules. Furthermore, the supported HPAs can be transformed into metal oxides under controllable thermal treatment conditions. With high loading of HPAs, nanowires and even three-dimensional nano-structured metal oxide(s) can be fabricated by reverse replication of the channels of mesoporous silica. This is a powerful way to prepare nanostructured metal oxides with uniform morphology. Here we review the applications of mesoporous silica supported HPAs in heterogeneous catalysis and the preparation of nanostructured metal oxides.

## 2. Heterogeneous Catalysis by HPAs Supported on Mesoporous Silicas

### 2.1. Impregnation of HPA on mesoporous silica

A mesoporous silica supported HPA may be easily obtained by the wet impregnation technique [32]. After stirring the mixture of mesoporous silica and HPA in water/methanol, HPA molecules can be introduced in the channels through the interaction between Si-OH and HPA molecules [33]. The supported HPA materials have high surface area and large pore size. HPA is highly dispersed without formation of crystal phases, even at quite high HPA loading. The highly dispersed HPA exhibits high density of Brönsted acid sites in the channels [34,35]. Mesoporous silica supported HPAs have been demonstrated to be efficient catalysts in heterogeneous reactions, such as Friedel-Crafts, dehydration, aldol condensation and oxidation reactions, as well as acetalization, Beckmann rearrangement and syntheses of heterocyclic compounds.

#### 2.1.1. Friedel-Crafts and related reactions

The conventional catalysts for the Friedel-Crafts reaction in homogeneous systems are AlCl_3_, H_2_SO_4_ and BF_3_, which cause serious environmental and operational problems, such as high toxicity, corrosion, difficulty in separation and recycling, *etc.* HPAs have shown their advantages over the traditional ones as eco-friendly homogeneous catalysts in early research. Recently, mesoporous silica supported HPAs also have been proven to be efficient catalysts for Friedel-Crafts reactions in heterogeneous system. 

**Scheme 1:**
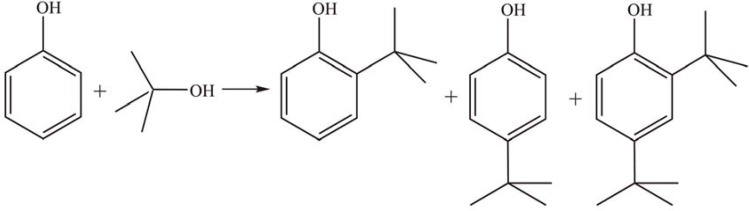
Butylation of phenol with *tert*-butanol over supported PTA.

Murugesan *et al.* [36,37,38] prepared a series of supported HPAs by impregnation of phosphotungstic acid (PTA) on mesoporous aluminophosphate (AlPO), Al-MCM-41, and SBA-15. Their catalytic activities in *t*-butylation of phenol with *tert*-butanol (Scheme 1) in the temperature range of 190–200 °C were studied. The products obtained were *o*-*tert*-butyl phenol, *p*-*tert*-butyl phenol, and *o*,*p*-di-*tert*-butyl phenol or *tert*-butyl phenyl ether, with high selectivity towards *p*-*tert*-butyl phenol (78.0–83.7%). The acidity of the catalysts can be controlled by adjusting the loading amount of HPAs and is directly correlated to the density of Brönsted acid sites. The optimal catalytic activity was achieved over the catalyst with the highest Brönsted acid density.

**Scheme 2:**
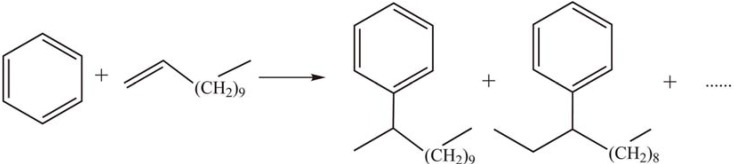
Alkylation of 1-dodecene with benzene over supported PTA.

Mesoporous silica supported HPAs have also been applied in the synthesis of monoalkylbenzenes attached to long chain olefins. Wang *et al.* [39] found that the SBA-15 supported PTA catalyst exhibited much higher catalytic activity, selectivity and stability than HY zeolite in alkylation of 1-dodecene with benzene at the reaction temperature of 80 °C (Scheme 2). The reaction involves a carbonium ion mechanism which results in the formation of five monoalkylbenzene isomers among the products. The selectivity of 2-phenyldodecane (37.3%) was higher than those for other isomers at 89.7% conversion of 1-dodecene. Llanos *et al.* [40] investigated the catalytic activities of PTA/MCM-41(Si) and PTA/MCM-41(Si/Al) in the alkylation of toluene with 1-dodecene. The MCM-41/(Si) supported HPAs have higher acid concentration than the MCM-41/(Si/Al) supported ones due to the strong interaction between PTA and the Al sites of the MCM-41/(Si/Al) supports. The activities also depend on the acid concentration. The selectivity of monoalkylated products reached 100% at 28% conversion of 1-dodecene using 60% PTA/MCM-41(Si) as the catalyst.

**Scheme 3:**
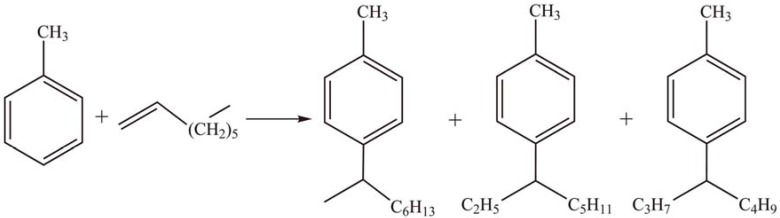
Alkylation of toluene with 1-octene over supported Keggin-type HPAs.

Xu *et al.* [41] reported the liquid phase alkylation of toluene with 1-octene (Scheme 3) catalyzed by bulk and MCM-41-supported Keggin-type HPAs, such as PTA, silicotungstic acid (STA), and phosphomolybdic acid (PMA). All supported catalysts, especially MCM-41 supported STA and PTA, exhibited higher activity than the bulk HPAs. The conversion of 1-octene was 100% and selectivity for monoalkylation products was 99.9% after 2 h of reaction at 120 °C over STA/MCM-41. The catalysts retained their catalytic activity for five runs, indicating only slight leaching of HPAs in a low polarity solvent.

**Scheme 4:**
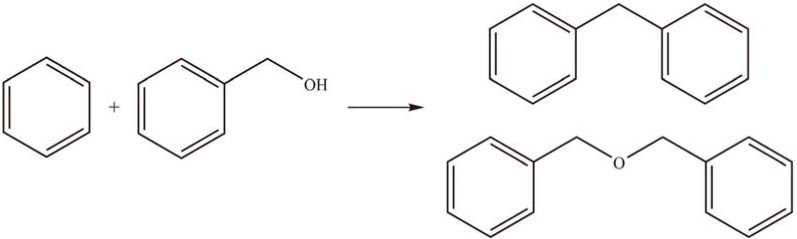
Benzylation of benzene with benzyl alcohol over supported Keggin-type HPAs.

Sugi *et al.* [42] investigated the benzylation of benzene and substituted aromatics with benzyl alcohol (BnOH) (Scheme 4) using a series of HPAs supported on MCM-41, FSM-16, and SBA-15. The conversion of BnOH and the yield of diphenylmethane decreased in the order of PTA > STA > PMA. This result showed that the activities were related to the acidic strength of the HPAs. The conversion of BnOH increased with temperature and reached 100% at 90 °C. The yield of diphenylmethane reached 80% when 50 wt % PTA/MCM-41 was used as a heterogeneous catalyst. The turnover number (TON) of PTA/MCM-41 after 1 h reaction time was 4–6 times higher than that of unsupported PTA. The catalytic activity gradually decreased from 86% to 75% after five reaction cycles.

**Scheme 5:**
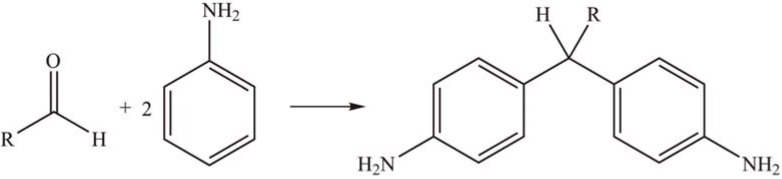
Reaction of aniline with aromatic aldehyde over supported PTA.

The catalytic activity of bulk PTA and supported PTA in the reaction of aniline with various aromatic aldehydes under liquid-phase condition was studied (Scheme 5). The activity of the supported PTA was slightly lower than the bulk one, but much higher than that of zeolites. The reusability and stability of the supported catalysts were higher in non-polar solvents than in moderately polar solvents [43].

Wang *et al.* [44] reported isopropylation of naphthalene with isopropanol over SBA-15-supported PTA. The PTA/SBA-15 showed much higher conversion and selectivity to diisopropylnaphthalene, *β*-isopropylnaphthalene, and *β*, *β*-products than that of pure acid. The activity of 50 wt % PTA/SBA-15 was comparable with the USY catalyst, much higher than bulk HPA and H-mordenite catalysts. It was revealed that the conversion decreased drastically from 84.3% to less than 20% after the fourth reaction cycle due to the leaching of PTA from the support caused by the polar isopropanol and water in this reaction.

#### 2.1.2. Dehydration reactions

**Scheme 6:**
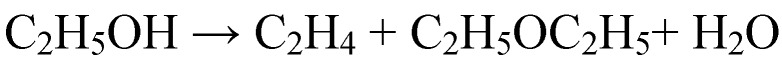
Dehydration of ethanol to form ethylene and diethylether over supported STA.

MCM-41 and MCM-41(Si/Al)-supported STA can catalyze the dehydration of ethanol to form ethylene and diethylether (DEE) (Scheme 6). Ethylene yield showed an increasing trend with temperature, reaching to *ca*. 100% above 250 °C and DEE was formed at lower temperatures, reaching to a yield of about 70% at 180 °C over STA/MCM-41. In contrast, with increasing temperature the yield of DEE passed through a maximum at *ca.* 220 °C and the ethylene yield reached 100% at 220 °C for the STA/MCM-41(Si/Al). The difference should be attributed to the effect of Lewis acid of the MCM-41(Si/Al) support. At low temperature, the formation of DEE and ethylene are probably taking place in parallel pathways involving Brönsted and Lewis acids, respectively. At high temperature, the main product is ethylene which is formed by the decomposition of DEE [45].

**Scheme 7:**
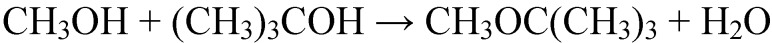
Dehydration of MeOH and *t*-BuOH to form MTBE over supported PTA.

MCM-41-supported PTA displays high activity in the gas phase preparation of methyl *tert*-butylether (MTBE) (Scheme 7). The 50 wt % PTA/MCM-41 shows nearly the same activity as the commercial Amberlyst-15 catalyst. The catalyst is stable, without any deactivation being observed after up to 100 h of reaction time [46].

**Scheme 8:**
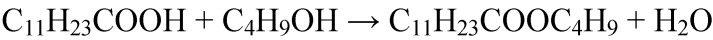
Esterification of lauric acid with butanol over supported PTA.

The MCM-41-supported PTA also shows higher conversion than the parent PTA for esterification of the long-chain lauric acid with butanol (Scheme 8). The catalytic activity is slightly higher than the commercial Amberlyst-15 in the first two reaction cycles but decreased significantly due to the leaching of the PTA [47].

#### 2.1.3. Condensation reactions

**Scheme 9:**
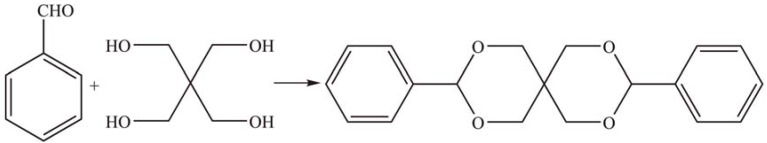
Acetalization of carbonyl compounds with pentaerythritol over supported PTA.

Supported HPAs may be applied in catalytic condensation reactions. Pandurangan *et al.* [48] investigated the acetalization of carbonyl compounds with pentaerythritol catalyzed by PTA/MCM-41 at reflux temperatures (Scheme 9). The activity of 15 wt % PTA/MCM-41 was comparable to that of homogeneous PTA catalyst. When loading the PTA on the as-prepared MCM-41 (with template in the channels), the activity decreased significantly. This phenomenon indicates that the reaction occurs mainly within the pores of the catalyst.

#### 2.1.4. Other reactions

**Scheme 10:**
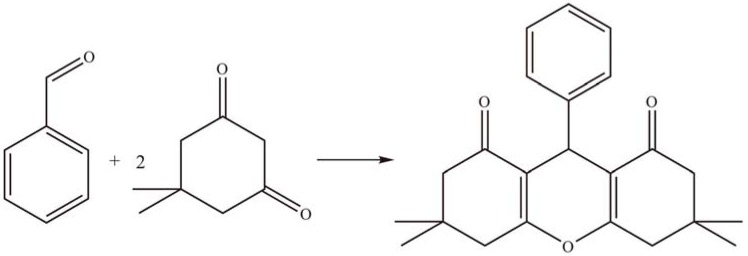
Condensation of dimedone and aromatic aldehyde over supported PTA.

Pandurangan *et al.* [49] synthesized a series of xanthenedione derivatives by condensation of dimedone and various aromatic aldehydes using MCM-41-supported PTA as solid acid catalyst (Scheme 10). The yield of xanthenedione reached 94% in ethanol at 90 °C. There was a significant loss of HPA species for the first cycle, but the loss diminished for later cycles and the product yield was gradually decreased to 84%.

**Scheme 11:**
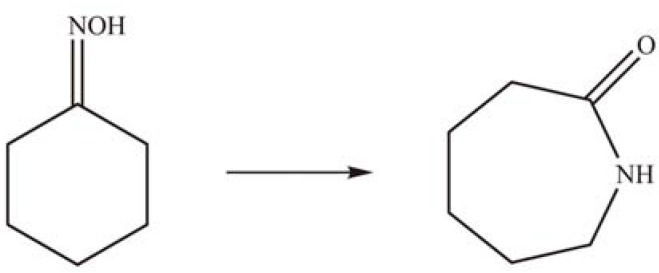
Beckmann rearrangement of cyclohexanone oxime to *ε*-caprolactam over supported PTA.

Narayanan *et al.* [50] reported the vapor-phase Beckmann rearrangement of cyclohexanone oxime to *ε*-caprolactam on a supported PTA catalyst (Scheme 11). Between 300 and 325 °C, a conversion of >99% with *ε*-caprolactam selectivity of 75% was achieved on 30 wt % PTA/SiMCM-41. The conversion and selectivity were much higher than the early reported homogeneous catalysts [51].

**Scheme 12:**
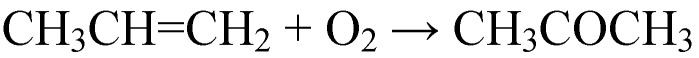
Oxidation of propylene to acetone over supported transition metal salts of H_5_PMo_10_V_2_O_40_.

Liu and co-workers [52] introduced the transition metal salts of 10-molybdo-2-vanadophosphoric acid (H_5_PMo_10_V_2_O_40_) on HMS, and the resulting materials were used as catalysts for the selective oxidation of propylene to acetone with molecular oxygen (Scheme 12). By supporting CuH_3_PMo_10_V_2_O_40_ on HMS, the conversion increased to 17.8% and the selectivity for acetone increased to 84.2% at 423 K.

### 2.2. Grafting of HPAs on amino-functionalized mesoporous silica

**Scheme 13:**
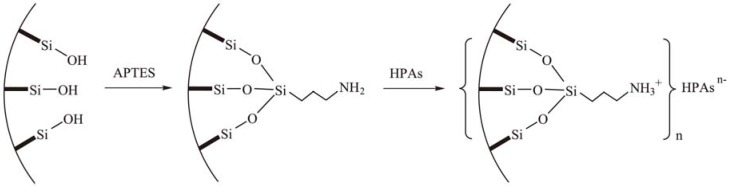
The functionalization of mesoporous silica with APTES and the anchoring of HPAs.

The wet impregnation technique is a feasible method to introduce HPAs onto silica supports. Through this method, HPAs can be highly dispersed on the support with large surface area which improves their catalytic performances. However, there is no strong chemical interaction between HPAs and the silica support, hence, HPAs tend to leach out of the supports, especially in polar reaction media, leading to deactivation of the catalysts [53]. In order to avoid leaching, the grafting method has been adopted to immobilize HPA on the supports. As reported by Vansant and coworkers [54], the silica surface may be modified with aminoalkoxysilanes, which provides functional amino groups on the silica surface. HPAs and the basic amine groups form the ≡Si(CH_2_)_n_NH_3_·HPA salt [55], which results the strong anchoring of HPAs and prevents the leaching of HPAs [56]. This type of heterogeneous catalysts (Scheme 13) are solvent-tolerant in the reactions involving polar reaction media, even photocatalytic degradation of organic pollutants in water.

#### 2.2.1. Dehydration reactions

**Scheme 14:**
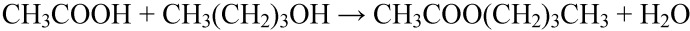
Esterification of *n*-butanol with acetic acid over grafted Keggin and Preyssler type HPAs.

Keggin and Preyssler type HPAs were grafted on 3-aminopropyltriethoxy silane (APTES)-modified SBA-15 and MCM-41 and used as heterogeneous catalysts in the esterification of *n*-butanol with acetic acid (Scheme 14). The conversion of *n*-butanol over the grafted HPA catalysts was lower than the corresponding ones prepared by impregnation method due to the low loading of HPA in the former. However, after several reaction cycles the grafted catalysts only deactivated slightly although water was formed in the dehydration reactions [57,58]. In contrast, for the impregnated catalysts serious leaching was observed.

**Scheme 15:**
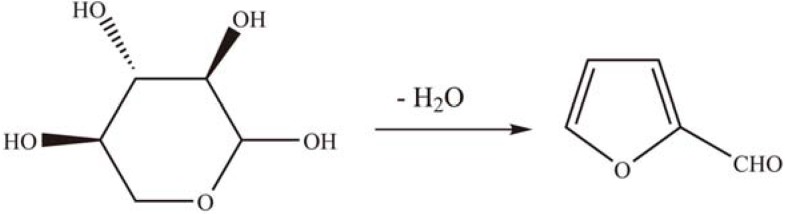
Liquid phase conversion of d-xylose to furfural over grafted PTA.

PTA immobilized on APTES-functionalized mesoporous silica showed dehydration activity in the liquid phase conversion of D-xylose to furfural (Scheme 15). The catalyst exhibited higher activity for D-xylose dehydration than the unsupported PTA and was comparable with H_2_SO_4_ (58%) in terms of furfural yield after 4 h reaction under similar conditions [59].

#### 2.2.2. Oxidation reactions

**Scheme 16:**
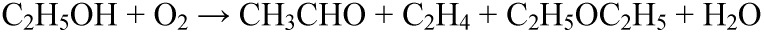
Vapor-phase ethanol conversion reaction over grafted PMA.

Song *et al.* [60,61] investigated the vapor-phase ethanol conversion reaction catalyzed by APTES- modified mesoporous MCF supported PMA (Scheme 16). The PMA/APTES/MCF catalyst showed a remarkably enhanced ethanol conversion compared to the bulk PMA catalyst. The interaction between the PMA and amino groups suppressed the acid catalytic activity of PMA to form ethylene and diethylether, leading to a high selectivity to the oxidation catalytic product (acetaldehyde).

**Scheme 17:**
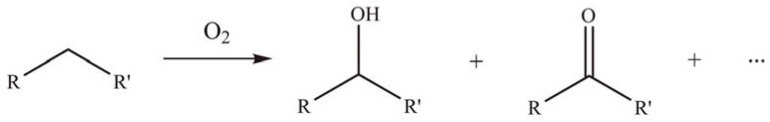
Oxidation of long-chain *n*-hexadecane over grafted HPAs.

Richards and co-worker [62,63] immobilized [Fe_4_(H_2_O)_10_(*β*-XW_9_O_33_)_2_]^n-^ (n = 6, X = As^III^, Sb^III^; n = 4, X = Se^IV^, Te^IV^) on APTES-modified SBA-15. The catalysts showed excellent catalytic performance for solvent-free aerobic oxidation of long-chain *n*-hexadecane using air as the oxidant under ambient conditions (Scheme 17). The conversion of *n*-hexadecane was the highest over Fe_4_Se_2_W_18_/APTES/SBA-15 (*ca*. 18%), whereas the selectivities to C16 ketones (*ca*. 50%) and C16 alcohols (*ca*. 28%) were similar for all Fe_4_X_2_W_18_/APTES/SBA-15 catalysts. The large polyanion [Cu_20_Cl(OH)_24_(H_2_O)_12_(P_8_W_48_O_184_)]^25-^ (Cu_20_) immobilized on the APTES/SBA-15 also showed activity in aerobic oxidation of *n*-hexadecane. The heterogeneous Cu_20_/APTES/SBA-15 catalyst was very active, with 29.4% conversion of *n*-hexadecane and 52% selectivity to ketones, with an exceptionally high turnover-frequency of 20,000 h^-1^. The catalysts were easily recycled by filtration without loss of activity.

**Scheme 18:**
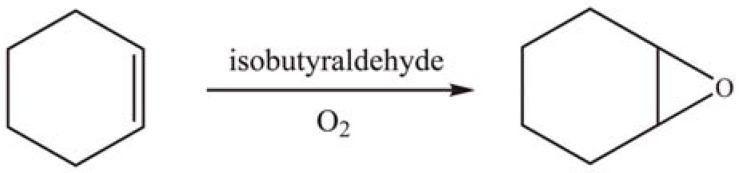
Oxidation of cyclohexene over grafted PW_11_Co and SiW_9_Co_3_.

The APTES modified MCM-41 supported [Co^II^(H_2_O)PW_11_O_39_]^5-^ (PW_11_Co) and [{Co^II^(H_2_O)}_3_SiW_9_O_37_]^10-^ (SiW_9_Co_3_) were applied in the oxidation of cyclohexene using molecular oxygen as oxidant in the presence of isobutyraldehyde (Scheme 18). SiW_9_Co_3_/APTES/MCM-41 exhibited significantly higher activity to produce cyclohexene oxide than the monosubstituted PW_11_Co/APTES/MCM-41 due to more Co^II^ centers in the former available for the reaction. The selectivity for cyclohexene oxide approached 100% [64].

**Scheme 19:**
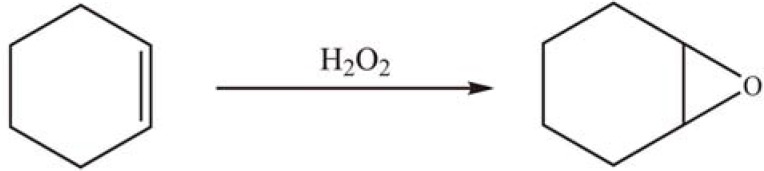
Oxidation of cyclohexene over grafted K_11_[RE(PW_11_O_39_)_2_].

Rare earth metal sandwiched Keggin-type heteropolyoxometalates, K_11_[RE(PW_11_O_39_)_2_] (REPW_11_, RE = La, Ce, Pr, Nd, Sm, Eu, Dy and Y), were anchored onto aminosilylated SBA-15 and the resulting REPW_11_/APTES/SBA-15 materials were applied in the oxidation of cyclohexene using H_2_O_2_ as oxidant (Scheme 19). The catalysts showed higher catalytic efficiency per polyoxometalate (POM) unit than the bulk POM. The REPW_11_ can be strongly anchored onto SBA-15. The leaching of REPW_11_ species was negligible in reaction cycles [65,66]. These results suggest that the anchoring of rare-earth metal substituted POM on the grafted silica surface is possibly due to the coordination of metal ions with amino-groups.

APTES-functionalized SBA-15-supported H_4_PMo_11_VO_40_, H_5_PMo_10_V_2_O_40_ and H_6_PMo_9_V_3_O_40_ are also efficient catalysts in the oxidation of acetaldehyde by molecular oxygen under ambient conditions. The acetaldehyde conversion approached 71.7% at 20 °C [67]. The oxidation of norbornene, cyclooctene, cyclohexene and styrene with aqueous H_2_O_2_ has been carried out with the immobilized H_4_PMo_11_VO_40_ catalyst (PMo_11_V/APTES/SBA-15). The selectivities of the desired products were higher than with the bulk PMo_11_V [68].

**Scheme 20:**
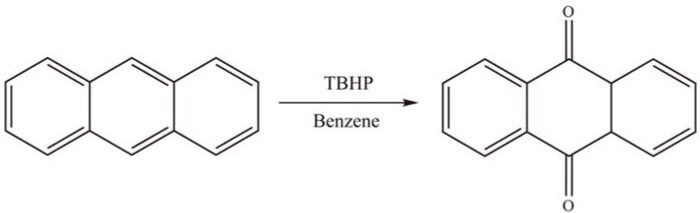
Oxidation of anthracene over grafted H_3__+x_PMo_12__-x_V_x_O_40_.

Halligudi *et al.* [69] reported the immobilization of H_3__+x_PMo_12__-x_V_x_O_40_ (x = 0–3) onto mesoporous silicas such as MCM-41, MCM-48, and SBA-15, through APTES linkers. These heterogeneous catalysts were tested in the liquid-phase oxidation of anthracene (AN) with *tert*-butylhydroperoxide oxidant in benzene (Scheme 20). Among the catalysts, H_5_PMo_10_V_2_O_40_ immobilized onto amine-functionalized SBA-15 gave 60% AN conversion with 100% selectivity for anthraquinone. The high activity of APTES/SBA-15 supported catalyst is attributed to its lower diffusion constant than that of MCM-41 and MCM-48.

**Scheme 21:**
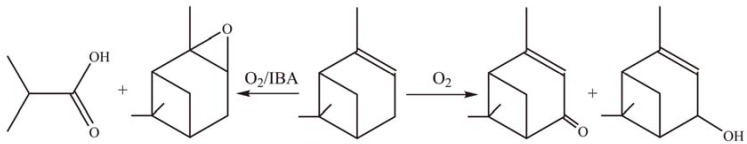
Autoxidation and co-oxidation of *α*-pinene over grafted Co-POM.

[Bu_4_N]_4_H[PW_11_Co(H_2_O)O_39_] (Co-POM) was supported on APTES-modified SBA-15 and MCF to catalyze the autoxidation and co-oxidation of *α*-pinene (Scheme 21). In the autoxidation process, the formation of the allylic oxidation products, verbenol and verbenone with 60–70% selectivity at *ca.* 20% conversion was achieved over the Co-POM/APTES/SBA-15 catalysts. In the case of *α*-pinene and isobutyraldehyde (IBA) co-oxidation, the amino groups on the support neutralized the formation of carboxylic acid, leading to the high selectivity of *α*-pinene epoxide. The Co-POM/APTES/MCF was the most selective catalyst and the selectivity to *α*-pinene epoxide reached 94% at 96% conversion after 2 h reaction time [70].

#### 2.2.3. Photocatalytic degradation

The amino-functionalized mesoporous silica HPAs may be applied in photocatalytic degradation of organic pollutants, such as rhodamine B, hexachlorobenzene and methylparathion. The catalytic activities of supported HPAs are better than those of the bulk ones. The major advantage of the supported HPAs over homogeneous catalysts is not their reactivity, but rather the ease of separation and recovery of the catalysts from the solutions. Only limited leaching of HPAs was detected. The catalysts may be recycled several times without significant changes in activity [71,72].

### 2.3. Entrapping of HPAs in porous silica matrix via a sol-gel method

HPAs can be incorporated into the silica matrix through sol-gel processes. These are a simple route to prepare silica-supported HPA materials in comparison with the grafting methods. HPA can be immobilized into the silica matrix strongly by electrostatic or complex interactions between the silica support and the HPA. The obtained HPA-in-SiO_2_ materials are insoluble, highly thermal stable and readily separable. HPA in silica matrix exhibits resistance to leaching, even after hot water extraction at 373 K. It was observed that the incorporation of the HPAs molecules lead to the decrease of the pore size and surface area. They have been proved to be efficient heterogeneous catalysts for dehydration [73,74,75,76,77,78,79,80] and oxidation reactions [81,82,83].

#### 2.3.1. Dehydration reactions

Qi *et al.* introduced PTA into the mesoporous molecule sieve SBA-15 by a sol-gel technique [74]. The surface areas of the PTA-in-SBA-15 samples were larger than the same PTA-containing samples prepared by an impregnation method because the existence of considerable amount of PTA in the pore walls of the PTA-in-SBA-15 sample. In the esterification reactions between alcohols and acetic acid, the conversions over the catalyst prepared by the sol-gel method was comparatively constant (40%) after a sharp decrease at the first reaction cycle. In contrast, the impregnated catalyst lost its activity rapidly due to the easy leaching of HPAs in the reaction system. Castanheiro *et al.* [79] immobilized PMA and STA on silica through a sol-gel technique. It was observed that the catalytic activity for esterification of fatty acid with methanol decreased in the order of PTA-silica > STA-silica > PMA-silica, indicating that their activities depended on the acid strength of the HPA precursors. He *et al.* [75] incorporated PTA in SBA-15 matrix and studied its activity in the dehydration of acetic acid. The selectivity to acetic anhydride reached 96% at 55% conversion.

Qiu *et al.* [76,77] reported the synthesis of a series of PTA- and PMA-containing silica matrices. Their activities were comparable to bulk HPA in catalytic reactions involving small molecules (cumene cracking and esterification of ethanol with acetic acid) and bulky molecules (1,3,5-triisopropylbenzene cracking and esterification of benzoic acid with *tert*-butanol). The catalysts could be separated and recycled easily, even in polar solvents.

**Scheme 22:**
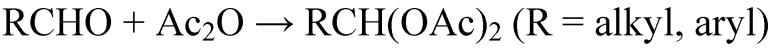
Dehydration of aldehydes to form 1,1-diacetates over PTA-incorporated mesoporous silica matrix.

Wang *et al.* [78] reported the preparation of 1,1-diacetates from aldehydes under solvent-free conditions at room temperature using PTA-incorporated mesoporous silica matrix as catalyst (Scheme 22). The catalyst displayed high conversion of aldehydes to acylals. The yields of 1,1-diacetates reached to as high as 89–98%, based on different substrates.

#### 2.3.2. Oxidation reactions

**Scheme 23:**
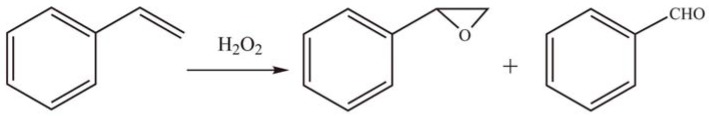
Oxidation of styrene over the SBA-15 incorporated [(*n*-C_4_H_9_)_4_N]_4_[*γ*-SiW_10_O_34_(H_2_O)_2_].

Guo *et al.* [82] reported the catalytic oxidation of styrene with H_2_O_2_ by the SBA-15 incorporated divacant Keggin [(*n*-C_4_H_9_)_4_N]_4_[*γ*-SiW_10_O_34_(H_2_O)_2_] (Scheme 23). The activity of the catalyst for the catalytic oxidation of styrene is solvent dependent. The activity decreased in the order of acetonitrile > acetone > toluene. The selectivity to styrene oxide was 77.1% at 27.7% conversion.

**Scheme 24:**
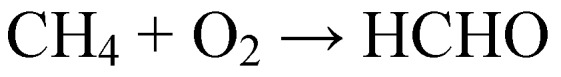
Oxidation of methane to formaldehyde over P-Mo-V mixed oxide-incorporating mesoporous silica.

He and co-workers [81] reported a series of P-Mo-V mixed oxide-incorporating mesoporous silica catalysts. The catalysts were prepared by a templating method using H_5_PMo_10_V_2_O_40_ as precursor and co-acid, followed by calcination at 873 K. The P:Mo:V molar ratio of 1:10:2 in the catalysts indicated that there was no significant composition change from the HPAs precursor to the mixed oxide and catalysts with defined V/Mo/P ratio may be obtained by choosing suitable POM precursor. The catalysts showed good activity in the selective oxidation of methane to formaldehyde (Scheme 24).

## 3. Preparation of Nanostructured Metal Oxides Based on HPAs as Precursors and Mesoporous Silicas as Hard Templates

Due to the diversity of structure and property, transition metal oxides, such as WO_3_, V_2_O_5_, and MoO_3_, have wide applications in the fields of catalysis and functional materials. [84,85,86]. When the particle size in one or more dimensions is in the nanometer range, the oxides will show special properties. Compared with the metal nanomaterials, the crystallization of the nanostructured metal oxides is difficult.

Nanocasting, using mesoporous silica as template, has brought about great possibilities in preparing nanostructured materials. Ryoo and co-workers [87] reported the first fabrication of mesoscopically ordered nanoporous (or mesoporous) carbon molecular sieves by carbonizing sucrose inside the pores of mesoporous silica MCM-48. Different from the previous nanocasting strategy using organic amines or surfactants as templates, this method uses highly ordered mesoporous silica as the hard template. Mesoporous silicas with pore sizes of 2–50 nm and high surface area serve as nanoscaled reactors for preparation of various nanomaterials with controllable size and shape. Besides MCM-48, SBA-15 [25], SBA-16 [26], FDU-5 [88] or KIT-6 [89] with hexagonal or cubic pore structures can also be used as templates for duplication of nanostuctures.

The filling of precursors into the hard template channels is a key process which affects the final quality of nanostructures. The precursor may be incorporated into the channels of mesoporous silica by various methods, such as impregnation, sorption, and grafting. Upon thermal treatment, the precursor is decomposed and crystallized inside the channels. By a simple impregnation method, Au [90], Ag [91,92], and Pt [93] nanowires were prepared through thermal decomposition of precursors in mesopores of SBA-15. Using this method and metal nitrates as precursors, Tian *et al.* [94] obtained a number of metal oxide nano-arrays. However, this method has certain drawbacks, *i.e.* the precursors and the templates are not closely integrated, leading to relatively poor filling of mesopores and formation of imperfect nanostructured materials.

In order to improve the filling of precursor within mesoporous silica, the grafting method was applied to strengthen the interaction between precursors and templates. The channel surface of mesoporous silica was modified by aminoalkoxysilanes, such as 3-aminopropyltriethoxy silane (APTES) [54]. The strong interaction between the amino groups and the protons may firmly anchor HPA molecules inside the channels of mesoporous silica with high loading. The nanostructured early transition metal oxide can be obtained through decomposition of HPA under controlled thermal treatment. According to this route, a series of nanostructured metal oxides have been successfully prepared using amino-functionalized mesoporous silica SBA-15 as hard template. In addition, in this method transition metal salts may be used as precursors.

### 3.1. Preparation of one-dimensional metal oxide nanowires based on HPAs

**Figure 1:**
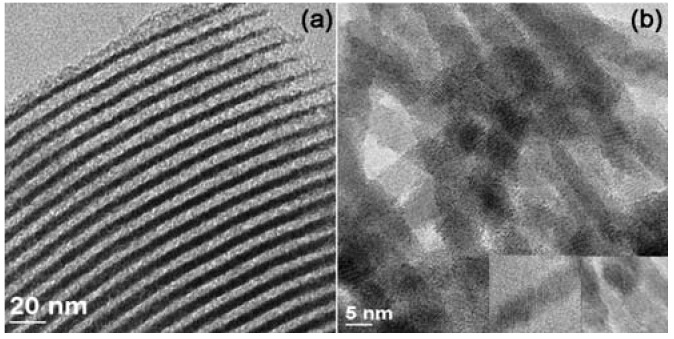
TEM images of nanowires: (a) V_2_O_5_ in SBA-15 [95] (Reproduced by the permission of Wiley-VCH); (b) WO_3_ without hard template SBA-15 [97] (Reproduced by the permission of Elsevier).

With the APTES-functionalized SBA-15 as hard template and HPAs as precursors, one-dimensional (1D) V_2_O_5__,_ MoO_3_-V_2_O_5_, and WO_3_ nanowires were obtained. The 1D V_2_O_5_ [95] and MoO_3_-V_2_O_5_ nanowires [96] were prepared by introducing isopolyacid, decavanadoacid and Keggin-type molybdovanadophosphoric acids into the channels of amino-functionalized SBA-15, respectively, followed by thermal treatment. The TEM images of the nanowires indicate that the HPAs disperse uniformly inside the SBA-15 channels (Figure 1a). In the preparation of MoO_3_-V_2_O_5_ nanowires, it was found that the silica wall of SBA-15 was fragmented by penetration of sublimated MoO_3_ at high temperature (700 °C). These two types of nanowires cannot be kept intact during removal of the SBA-15 template by aqueous solution of NaOH or HF. 1D WO_3_ nanowires were also synthesized with H_3_PW_12_O_40_ as precursor [97]. The WO_3_ nanowires are stable after removal of the silica template by aqueous solution of HF (Figure 1b). From HRTEM study the prepared WO_3_ nanowires are single crystal and uniform in diameter.

### 3.2. Preparation of three-dimensional nanoporous metal oxides based on HPAs

**Figure 2:**
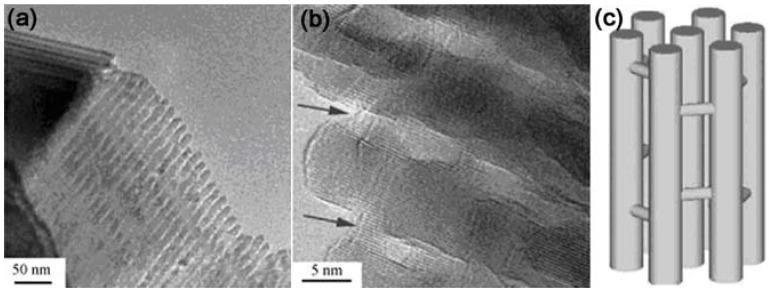
(a) TEM image of the 3D Cr_2_O_3_ mesoporous single crystals. (b) TEM image with higher magnification showing the ordering of the Cr_2_O_3_ nanorods. Two arrows indicate the bridges. (c) Schematic drawing of the structure of 3D Cr_2_O_3_ mesoporous single crystals [99]. (Reproduced by the permission of the Royal Society of Chemistry.)

When using the amino-functionalized SBA-15 as the hard template and Cr_2_O_7_^2-^ as the precursor, where Cr_2_O_7_^2-^ can considered as the smallest polyoxoanion [98], three-dimensional (3D) Cr_2_O_3_ mesoporous single crystals were obtained after thermal decomposition of Cr_2_O_7_^2-^ inside the mesoporous channels and removal of the silica template, [99]. The structure of the products is an array of hexagonally ordered nanorods linked by nanosized bridges to form a 3D open framework (Figure 2). The products can be regarded as single crystals that are carved with a honeycomb pattern. It is also noticeable that the diameter of the nanorods exceeds the pore diameter of SBA-15, indicating that the nanorods would continue to grow and eventually decompose the surrounding silica framework. It is even possible to form nonporous large single crystals when the temperature of the crystallization is too high and/or the time of the crystallization is too long. This phenomenon implies that the space between the nanorods may be tuned by controlling the experimental conditions. The pore size can be further enlarged during the crystallization. The formation of the bridges between the nanorods should contribute to the micropores on the SBA-15 silica wall. The dichromic acid is able to locate in these inter-channel micropores and became the bridges during thermal treatment.

**Figure 3:**
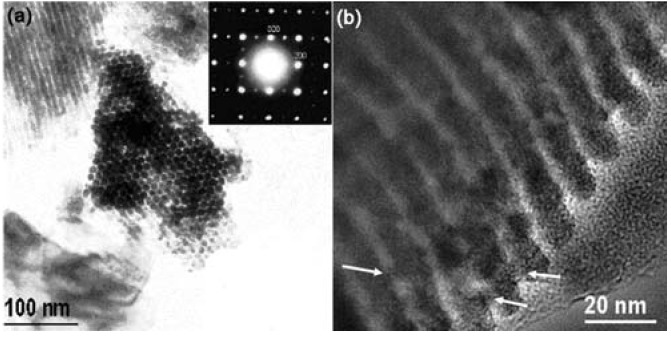
(a) TEM image of 3D mesoporous single crystals of WO_3_. The top right inset shows a SAED pattern from many nanorods when viewed down the [001] zone axis of the WO_3_ unit cell. (b) TEM image showing some bridges indicated by arrows [102]. (Reproduced by the permission of Elsevier.)

It is obvious that the size of the inter-channel micropores on the mesoporous silica wall is too small to accommodate large size HPA molecules. In order to prepare 3D meso-structured metal oxides based on HPAs, the amino-functionalized 3D SBA-15 [100,101] with larger micropores was chosen as hard template. H_3_PW_12_O_40_ was immobilized in the APTES/3D SBA-15 followed by thermal treatment to form WO_3_ [102]. After removal of the silica template, the morphology of the product is an array of hexagonally ordered nanorods linked by nanosized bridges to form a 3D open framework as an inverse replication of SBA-15 channels. Figure 3 shows typical TEM images of Si-free 3D mesoporous single crystals of WO_3_. The nanorods are connected by many short bridges, so that the original porous stucture of SBA-15 is maintained.

## 4. Conclusions

Mesoporous silica supported HPAs have attracted much attention in heterogeneous catalysis. The selected examples demonstrate their applications as heterogeneous catalysts with high catalytic activity, which results from the high dispersion of HPAs on the mesoporous silica with large pore size and high surface area. The mesoporous silica supported HPAs has a high mass transfer efficiency with better shape-selectivity than the bulk HPAs, which benefits the reactions involving large organic molecules. However, the leaching of HPA from the support in the reaction system containing polar solvents has limited their applications. The catalysts prepared using amino-functionalized mesoporous silica as support to anchor HPA or co-synthesis method through the sol-gel technique are more solvent-tolerant than the impregnated ones. Although the HPA catalysts prepared through these two modified methods show higher catalytic performance (specially much better recycle property), some limitations still exist, such as the decrease of the mass transfer efficiency in the amino-functionalized catalysts due to the reduction of pore size and the influence on synthesis and quality of mesoporous silica after adding HPAs in the sol-gel process. New progress is, therefore, expected to develop novel heterogeneous HPA catalysts with better catalytic performance. Furthermore, HPA can be used as the precursors to prepare nanostructured metal oxides using mesoporous silica as the hard template. The functionalization of the mesoporous silica is an efficient way to improve the filling of HPAs in the mesoporous channels. The crystalline nanostructured metal oxides can well replicate the channels of the mesoporous silica hard template.
